# Stability-Indicating HPLC Determination of Trandolapril in Bulk Drug and Pharmaceutical Dosage Forms

**DOI:** 10.1155/2015/820517

**Published:** 2015-01-31

**Authors:** Leena A. Al-Hawash, Ashok K. Shakya, Maher L. Saleem

**Affiliations:** ^1^Faculty of Pharmacy and Medical Sciences, Al-Ahliyya Amman University, P.O. Box 263, Amman 19328, Jordan; ^2^Arab Company for Drug Industries and Medical Appliances (ACDIMA), P.O. Box 925161, Amman 11190, Jordan; ^3^Faculty of Pharmacy, Middle East University, Airport Road, Amman 11831, Jordan

## Abstract

A rapid, simple, accurate, precise, economical, robust, and stability indicating reverse phase HPLC-PDA procedure has been developed and validated for the determination of trandolapril. The trandolapril was separated isocratically on Hypersil-Gold C18 column (250 mm × 4.6 mm, 5 *μ*m) with a mobile phase consisting of 50% acetonitrile and 50% water (containing 0.025% triethylamine, pH 3.0 ± 0.1), at 25 ± 2°C. Retention time of the drug was ~4.6 min. The eluted compounds were monitored and identified at 210 nm. The linearity of the method was excellent (*r* ^2^ > 0.9999) over the concentration range of 1–24 *μ*g/mL; the limit of detection (LOD) and limit of quantitation (LOQ) were 0.0566 *μ*g/mL and 0.1715 *μ*g/mL, respectively. The overall precision was less than 2%. Mean recovery of trandolapril was more than 99%; no interference was found from the component present in the preparation. Stability studies indicate that the drug was stable to sunlight and UV light. The drug gives 6 different oxidative products on exposure to hydrogen peroxide. Slight degradation was observed in acidic condition. Degradation was higher in the alkaline condition compared to other conditions. The robustness of the method was studied using factorial design experiment.

## 1. Introduction

**Figure 1:**
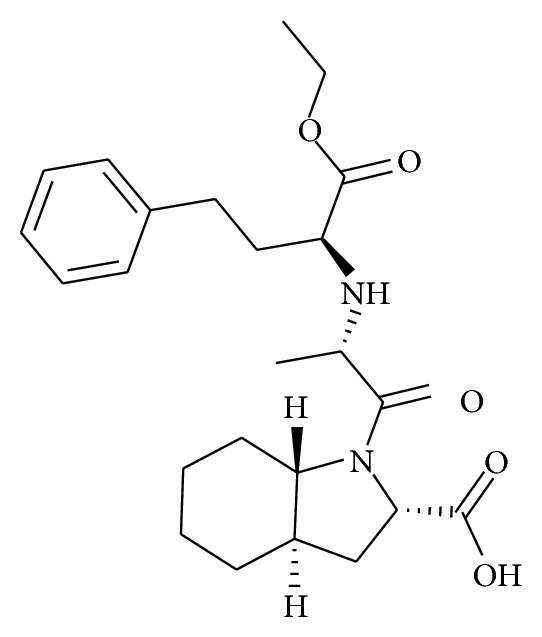
Structure of trandolapril.

Trandolapril (Figure 1) is a long-lasting angiotensin converting enzyme (ACE) inhibitor which was approved by the U.S. Food and Drug Administration for lowering blood pressure in doses up to 2 mg even after discontinuation of treatment [1]. It is also used for patient with evidence of Left Ventricular (LV) systolic dysfunction after Acute Myocardial Infarction (AMI). It can be given safely over a prolonged period of time [2]. Trandolapril is the international nonproprietary name of (2S, 3aR, 7aS)-1-[(2S)-2-{[(2S)-1-ethoxy-1-oxo-4-phenyl butan-2-yl]amino}propanoyl]–octahydro-1H–indole-2-carboxylic acid. Trandolapril is rapidly absorbed and metabolized to its biologically active diacid form, trandolaprilate, in liver which shows high lipophilicity compared to other ACE inhibitors. Determination of trandolapril alone has been analyzed less often than other drugs; indeed, only few methods involving amperometric biosensors [3], a potentiometric enantioselective membrane electrode [4], liquid chromatography tandem mass spectrometric [5], HPLC [6–13], HPTLC [14, 15], UV spectroscopy [16], and capillary electrophoresis [17] have been used. Trandolapril is official in British Pharmacopoeia and it is estimated by nonaqueous titration [18]. A few stability-indicating HPLC methods [11–13] have been reported, which provides variable level of degradation of trandolapril. Stability-indicating method reported by Manju Latha and Gowri Sankar [11] does not produce any degraded product in different stressed conditions, although it is well documented that trandolapril is susceptible to hydrolysis. Impurity profiles of trandolapril under stress (acidic and neutral) conditions studied by Dendeni et al. [12] require sophisticated LC-MS-MS instrument. Sahu et al. [13] have reported the validated stability-indicating method which can separate the hydrolytic degraded product of trandolapril. However none of the HPLC method reported the oxidative degraded product of trandolapril. International Conference on Harmonization (ICH) guidelines [19] require performance of stress tests on the drug substance, which can help to identify the likely degradation products. Moreover, validated stability-indicating methods should be applied in the stability studies [20] once they have demonstrated their suitability for their intended purpose. Thus, stability-indicating methods have to demonstrate that they are specific, which involves evaluating the drug in the presence of its degradation products. The aim of the present work was the development and validation of an HPLC stability-indicating method for determining trandolapril in its pharmaceutical form following ICH recommendations to achieve this goal; a stress study of the drug was performed in order to validate the stability-indicating power of the developed analytical method and to identify the key factors that will impact the stability of the drug product. The robustness of the developed method was studied using design of experiment utilising factorial design.

## 2. Experimental

### 2.1. Chemicals and Reagents

The reference sample of trandolapril (99.8%, TD0131207) was obtained as a gift from Hetero Pharmaceutical Ltd, Hyderabad, India. The marketed formulations of drug (Odrik hard gelatin capsules, strength 2 mg, manufactured by Abbott GmbH & Co KL, Germany) were purchased from local pharmacy. All reagents were of analytical grade unless stated otherwise. Reverse osmosis quality water (purified with a Milli-RO plus Milli-Q station Millipore Corp., USA) and HPLC quality water were used throughout. Acetonitrile and methanol (HPLC grade) were supplied by Panreac (Barcelona, Spain).

### 2.2. HPLC Instrumentation and Conditions

Analysis was performed with a Shimadzu Prominence liquid chromatograph equipped with LC-20AD UFLC quaternary solvent delivery system, SIL-20A Prominence autosampler having a universal loop injector of capacity 1–100 *μ*L, and an SPD-M20A diode array detector monitored between 200 and 350 nm and CBM-20A, Communication Bus module. The equipment was controlled by a Windows 7 based LC-Solution version 1.25 (2009-2010) work station software. Thermo Hypersil-Gold C-18 column (250 mm × 4.6 mm i.d.; 5 *μ*m) was used. The mobile phase was acetonitrile : water (50 : 50 v/v, containing triethylamine (TEA) 250 *μ*L/L, final pH adjusted to 3.0 with orthophosphoric acid (OPA)). Mobile phase was degassed using ultrasonic bath and sample solutions were filtered through 0.45 *μ*m filters prior to analysis. Mobile phase flow rate was 1.0 mL/min. All the analysis was carried out at 25 ± 2°C. Retention times, UV spectrum, and peak purity were used to identify trandolapril.

### 2.3. Preparation of Stock and Standards Solutions

#### 2.3.1. Stock and Working Solutions

Trandolapril (active pharmaceutical ingredient (API), equivalent to 100 mg of trandolapril) was weighed and transferred to 100 mL calibrated volumetric flask quantitatively. It was dissolved in acetonitrile (20 mL) with the aid of sonication. The final volume was made up to the mark with acetonitrile : water (50 : 50% v/v) to produce stock solution (1000 *μ*g/mL). Working solutions of trandolapril (25, 100, and 200 *μ*g/mL) were prepared using suitable aliquots of stock or intermediate solution. All the solutions were stored in refrigerator.

#### 2.3.2. Calibration Standards

Calibration standards were prepared freshly using the intermediate working solutions of trandolapril. Standard solution of concentrations 1, 2, 4, 8, 10, 12, 16, 20, and 24 *μ*g/mL was prepared. These solutions were analyzed immediately to avoid degradation and as per schedule.

#### 2.3.3. Quality Control Samples

Quality control samples at three concentrations (4, 12, and 22 *μ*g/mL) level were prepared separately as low quality control (LQC), medium quality control (MQC), and high quality control (HQC).

#### 2.3.4. Preparation of Sample for Assay

Average weight of twenty capsules content (each containing 2 mg trandolapril) was determined. A quantity of powder equivalent to 10 mg of trandolapril was weighed and transferred to 50 mL calibrated volumetric flask. Acetonitrile (10 mL) was added to the same flask and sonicated for 1 minute. The volume was made up to 50 mL with acetonitrile : water (50 : 50 v/v) solution. The theoretical concentration of the stock solution of trandolapril was 200 *μ*g/mL. The solution was filtered using 0.45 *μ*m nylon filter (Microsyringe filter). Appropriate dilutions were prepared for analysis.

### 2.4. Analytical Method Validation

#### 2.4.1. Linearity, Limit of Detection (LOD), and Limit of Quantitation (LOQ)

Appropriate volumes of trandolapril stock standard solution (1000 mg/mL) was diluted with mobile phase to produce concentrations of 1, 2, 4, 8, 10, 12, 16, 20, and 24 *μ*g/mL. Each day two different sets of calibration standard were independently prepared and analyzed. Six different calibration curves were prepared on three different days. To define the correlation between the response and concentration of trandolapril, the area was plotted against concentration of trandolapril with weighting factor *x*, 1/*x*, or 1/*x* ^2^. The method was evaluated by determination of the correlation coefficient and intercept values. The linear best fit line (weighting factor *x*) was used to measure the concentration of all samples throughout the batch. The acceptance criterion for each back calculated concentration was less than 2% from nominal values except for LOQ. Microsoft Office Excel 2007 was used for statistical analysis. The method was validated according to ICH guidelines of the validation of analytical methods [19, 20]. A 5% significance level was used for evaluation. LOD and LOQ were determined from the calibration function.

#### 2.4.2. System Suitability

System suitability parameters were tested with six replicate injections of the diluted sample of working standards (10 *μ*g/mL) at the start of the project and at the end of the project. The system suitability parameters were calculated using the internal features of LC-Solution software as per United States Pharmacopoeia [21]. The parameters were retention time, peak area and height, width at half peak height, tailing factor, efficiency, and height equivalent theoretical plate (HETP). System suitability was measured on the basis of precision (RSD). The precision, as measured by coefficient of variation was determined at each set's parameters and it should be less than 2% at the beginning of validation and at the end of validation.

#### 2.4.3. Precision

Precision was measured using triplicate determination of quality control samples of 4 *μ*g/mL (LQC), 12 *μ*g/mL (MQC), and 22 *μ*g/mL (HQC) of trandolapril, on three different occasions (0, 3, and 6 h) and different days. The precision (RSD) of the method was determined as intraday precision (repeatability) and intermediate precision. The intermediate precision was estimated from the RSD of the analysis of the samples prepared at the same concentration but on 3 different days at different concentration levels, while intraday precision was calculated by analyzing the same concentration during the same day at different time.

#### 2.4.4. Accuracy

Accuracy (as percentage recovery) was measured using replicate sample of trandolapril prepared using capsule matrix. Different samples (*n* = 3, at each level of 80%, 100%, and 120%) were prepared using capsules content (2 mg as 100%) and adding known quantity of trandolapril (at 80%–120% level). From these fortified samples, appropriate sample solutions were prepared and analyzed and the total amount recovered was calculated. Accuracy was calculated by comparing with true value. The concentrations were back-calculated by regression equation *y* = 21225*x* + 1303 (weighting factor *x*).

#### 2.4.5. Robustness

It is a measure of reproducibility of test results under normal, expected, operational condition from analyst to analyst. The robustness of the method was evaluated on the basis of precision, as measured by percent coefficient of variation (% CV or RSD), determined as each concentration level was required not to exceed 2%. Design of experiments (DOE) was used to study robustness of the method. A 2^4^ factorial design was used to test the robustness of chromatographic separation. The experimental design is useful for this kind of study as it facilitates the investigation of several parameters by reducing the number of experiments. Acetonitrile content of the mobile phase, volume of peak modifier, pH, and flow rate were investigated. Upper and lower limits are shown in Table 5. The experiments were run randomly and the selected responses were retention time (*T*_*r*_), tailing factor (*T*_*f*_), and area count.

#### 2.4.6. Stability Studies

Stress study like oxidative stress, alkaline stress, acidic stress, exposure to sunlight, and UV light (254 nm) were carried out using trandolapril raw material. Chromatograms were recorded in order to study the specificity of the method. The chromatograms of the samples were compared with those of control samples that were freshly prepared from the stock standard solution and without stress. All samples were analyzed in triplicate. The peak purity was checked using the tools of the LC-Solution software. This assessment was based on the comparison of spectra recorded during the elution of the peak. UV spectra and peak purity were used to assess purity of trandolapril.

*(1) Oxidative Stress.* Trandolapril (5 mg) was weighed accurately and transferred to 50 mL volumetric flask. 5 mL of 30% hydrogen peroxide was added to it. It was stirred for one hour, and then the contents were diluted to 50 mL with mobile phase. Replicate solutions of concentration 20 *μ*g/mL were prepared and chromatograms of these solutions were recorded and compared with the chromatograms obtained from the fresh solution of trandolapril having the same concentration and the chromatogram of the blank (solution containing only hydrogen peroxide).

*(2) Effect of Acid and Alkaline Media.* Trandolapril (5 mg) was weighed accurately and transferred to 50 mL volumetric flask. It was shaken for one hour with 5 mL of either 0.1 M hydrochloric acid (HCl) or 0.1 M sodium hydroxide (NaOH). After one hour the content was diluted to 50 mL with mobile phase. Replicate solutions of concentration 20 *μ*g/mL were prepared and chromatograms of these solutions were recorded and compared with the chromatograms obtained from the fresh solution of trandolapril having the same concentration and the chromatogram of the blank.

*(3) Effect of UV Light or Sunlight.* Trandolapril API (100 mg) was placed in an open watch glass and exposed to either UV-irradiation (~100 W/m^2^) or direct sunlight for two hours with occasionally shifting the content using stainless steel spatula. After two hours 10 mg of trandolapril was weighed and transferred to 10 mL volumetric flask. It was dissolved in mobile phase. 1 mL of the prepared solution was transferred to a 50 mL volumetric flask and diluted to the mark with mobile phase. Chromatograms were recorded and compared with the chromatogram of unexposed API. 

*(4) Stock Stability.* The stability of stock solution was evaluated at zero time and stored in the refrigerator (2–8°C). Samples were prepared and analysed at days 0, 7, 14, and 21.

## 3. Results and Discussion

### 3.1. Analytical Method Development

**Figure 2:**
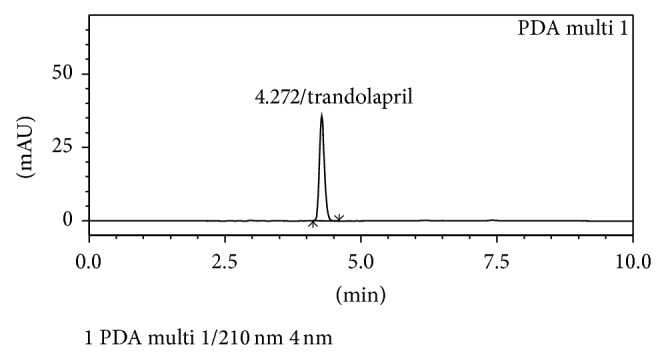
Representative chromatogram showing signal of trandolapril in the selected mobile phase.

The HPLC method was developed as a stability-indicating method to determine trandolapril in the presence of the possible degradation products (trandolaprilate) of the drug. Therefore, a retention time between 4 and 5 min was chosen for the drug since it allowed both a rapid determination of the drug, which is important for routine analysis, and a complete drug separation. During development step various mobile phases of water : methanol or water : acetonitrile (35 : 65, 40 : 60, 45 : 55, 50 : 50, 55 : 45, 60 : 40, and 65 : 35 v/v, with or without peak modifier, pH 3, 5, or 7) were tried and the responses were recorded. On the basis of responses and chromatographic parameters studied a mobile phase of acetonitrile: water (50 : 50 v/v, containing 0.25 mL/L TEA, final pH adjusted to 3.0 with OPA) was selected as suitable mobile phase, which can separate the drug and degraded products. Under these conditions the drug was eluted at ~4.3 min at ambient temperature (25 ± 2°C). The absorption maximum of the drug (*λ* 210 nm) was selected for detection, as there was no interference from excipients present in drug.  Figure 2 depicts the representative chromatogram obtained with the present method.

### 3.2. Method Validation

The method was validated with respect to parameters including linearity, LOQ, LOD, precision, accuracy, specificity, robustness, system suitability, and stability.

#### 3.2.1. Linearity

Different calibration curves (*n* = 6) were constructed for trandolapril was linear over the concentration range of 1–24 *μ*g/mL. Peak area of trandolapril was plotted versus trandolapril concentration and linear regression was performed using LC-Solution software and Microsoft Office Excel 2007. Different calibration curves were prepared on different days. The mean regression equation for trandolapril was found to be *y* = 21121  (±157.1)*x* + 1125  (±511.6)(weighting factor  *x*, Table 1). The regression coefficient was 0.9999 or higher. The linearity range reported in other methods ranged between 4 and 150 *μ*g/mL [6–13].

#### 3.2.2. LOD and LOQ

The LOD and LOQ values were 0.0566 and 0.1716 *μ*g/mL calculated using calibration curve as per ICH guideline. The LOD and LOQ reported by Sahu et al. [13] were 0.1 and 0.8 *μ*g/mL, based on signal to noise ratio method.

#### 3.2.3. Accuracy and Precision

The accuracy and precision of the analytical method were established across its linear range as indicated in the guideline. As shown from the data in Table 2, excellent recoveries (99.3 to 100.2%) were obtained at different added concentration level. The results obtained for the intraday and interday precision of the method were expressed as RSD values. As shown in the table, the intraday and interday RSD was < 2.0% for all concentrations tested in different situations studied (Table 3).

#### 3.2.4. Specificity

Specificity of the method was assessed by comparing the chromatograms obtained from capsule content and drug standards. The retention times of drug from standard solutions and from capsule content were identical and no coeluting peaks from the diluents were observed, indicating specific method for quantitative estimation of drug in the commercial formulation.

#### 3.2.5. System Suitability

System suitability parameters were studied with six replicates standard solution of the drug and the calculated parameters are within the acceptance criteria. The tailing factor, the number of theoretical plates, and HETP were in the acceptable limits (RSD less than 2%). The system suitability results are shown in Table 4.

#### 3.2.6. Robustness

**Figure 3:**
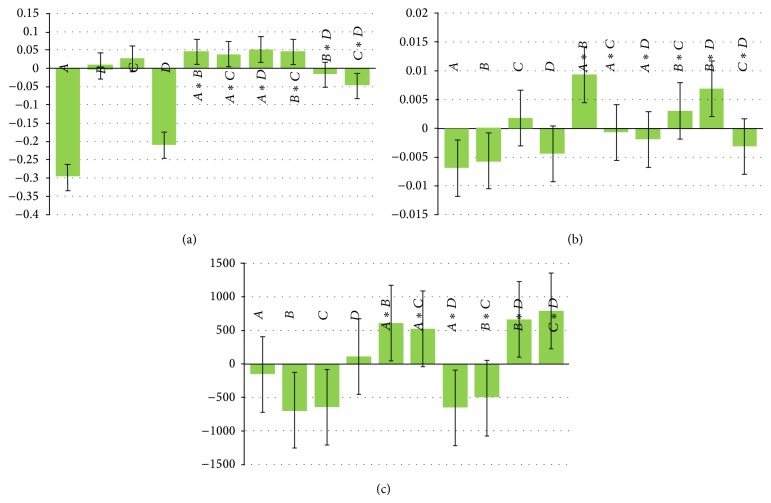
Scaled and centered coefficient of variation (%) of (a) retention time (*T*_*r*_), (b) tailing factor of drug (*T*_*f*_), and (c) area count.

Robustness of the methods was illustrated by getting the resolution factor and tailing factor, when mobile phase acetonitrile content (±5%), pH (±0.25 units), peak modifier (±0.05 mL/L), and flow rate (±0.1 mL/min) were deliberately varied. It was studied using factorial design experiment using Design Expert software version 8.0 (Stat Ease Inc, USA). The deliberate changes in the method do not affect the retention time, tailing factor, and area count for drug significantly. The scaled and centered coefficient plots for the above responses revealed that different parameters did not affect responses significantly, so that the developed method was considered rugged and robust. Results are presented in Figure 3 and Table 5.

#### 3.2.7. Stability Studies

**Figure 4:**
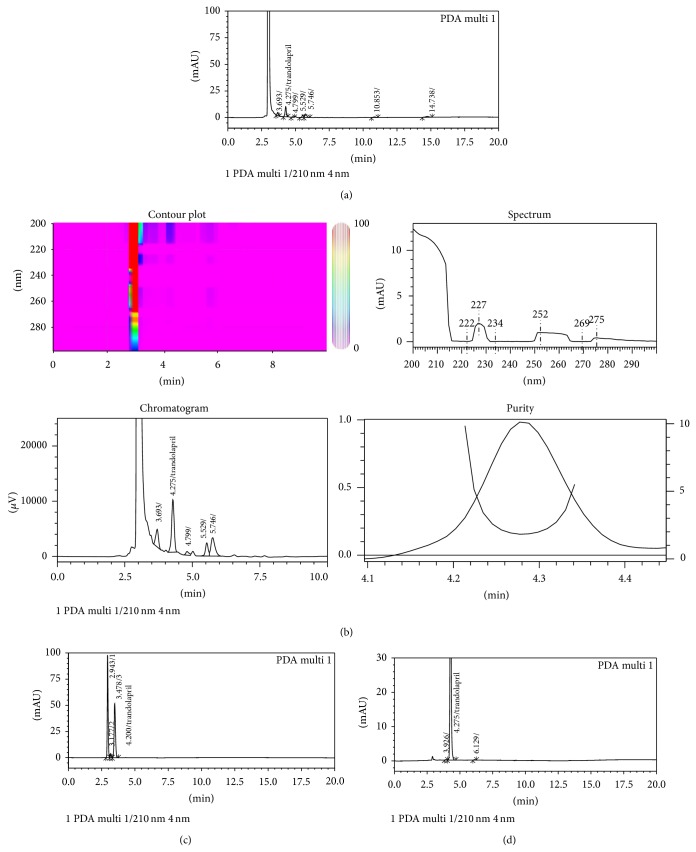
Typical HPLC chromatogram of trandolapril exposed to (a) oxidative stress, (b) enlarged view of chromatogram a showing contour plot, chromatogram, UV spectra, and peak purity, (c) alkaline stress, and (d) acidic stress condition showing degraded product.

The prepared stock and samples were stable up to 21 days when stored in refrigerator (2–8°C) and did not produce degraded compounds during experimental conditions. The peak purity was 0.985 or more during the validation studies. On exposure to hydrogen peroxide (30%), trandolapril produces six major degradation products having retention time 3.69, 4.79, 5.53, 5.75, 10.85, and 14.74 min. The percentage of unoxidized trandolapril was 40.9% (Figure 4(a), Table 6).  Figure 4(b) represents the chromatogram, peak purity, and UV spectra of the freshly prepared sample. After exposure to 0.1 M NaOH trandolapril gives 3 degradation product with retention time 2.943 (relative percentage 54.12%), 3.177 (2.22%), and 3.478 (43.66%). This indicates that alkaline conditions facilitate the conversion of trandolapril to different degraded compounds (Figure 4(c)). Results also indicate trandolapril degrades after exposure to 0.1 M HCl and forms degradation product having retention time 3.920 and 6.129 min. The percentage of undegraded trandolapril was 99.6% (Figure 4(d)). No degradation products were produced on exposure to the UV light or sunlight, which indicates that trandolapril has high stability under these stressed conditions.

LC-MS-MS identification of different degraded product and impurity profile of trandolapril under acidic and neutral conditions has been reported [12]. Sahu et al. [13] studied the hydrolytic decomposition of trandolapril (at a drug concentration of 2 mg/mL) under different conditions (acidic, alkaline, or neutral) at 80°C. The degradation of trandolapril was 50 and 65% under acidic and alkaline conditions, respectively. None of these studies (HPLC) reported the oxidative degradation of trandolapril. Our results reveal that the trandolapril is also susceptible to the oxidation. Vikas et al. [15] reported the two oxidative products of trandolapril using developed and validated HPTLC method.

#### 3.2.8. Assay

The proposed method was applied to the determination trandolapril in capsule formulations. The results of these assays yielded 99.2% (RSD = 0.89%) of label claimed. Low value of precision indicates that the method can be used precisely for the estimation of drug in formulations (Table 7).

## 4. Conclusion

A validated HPLC method has been developed for determination of trandolapril in formulations. The proposed method is simple, economical, accurate, precise, specific, robust, and stability-indicating. Robustness of chromatographic method was studied using design of experiments indicating robust and rugged method of analysis can be easily and conveniently adopted for the routine analysis of trandolapril in pharmaceutical dosage form and bulk drug.

## Figures and Tables

**Table 1:** Linearity of the present method.

Conc. *µ*g/mL	Mean area^*^	SD^*^	RSD
1	22,044	364.7	1.65
2	41,605	532.0	1.28
4	86,309	1194.8	1.38
8	172,070	1699.5	0.99
10	212,583	2283.2	1.07
12	255,392	2189.3	0.86
16	336,841	3371.1	1.00
20	424,233	3825.2	0.90
24	507,842	3534.7	0.70

Slope	21,121.5	157.1	
Intercept	1,125.3	511.6	
*r*	0.99996		

^*^Mean and SD of six determinations.

**Table 2:** Accuracy of the method.

Amount taken (mg)	Amount added		% Recovery (Mean ± SD) (*n* = 3)	% CV
	%	(mg)		
2	80	1.6	99.8 ± 0.29	0.29
2	100	2.0	99.3 ± 0.25	0.25
2	120	2.4	100.2 ± 0.24	0.24

**Table 3:** Precision study of the proposed method.

Concentration (*µ*g/mL)	Intraday precision		Interday precision	
	Conc. found Mean ± SD	% CV	Conc. found Mean ± SD	% CV
4	3.937 ± 0.068	1.74	4.008 ± 0.039	0.98
12	11.949 ± 0.088	0.74	12.011 ± 0.108	0.90
22	21.853 ± 0.102	0.47	21.731 ± 0.151	0.70

**Table 4:** System suitability parameters for trandolapril.

SN	*T*_*r*_ (min)	Area	Height	Conc. (*µ*g/mL)	Accuracy %	Tailing factor	Theoretical plate	USP width	HETP
Mean	4.28	210594	32575	10.06	100.6	1.12	8326	0.190	30.03
SD	0.003	737.05	118.93	0.03	0.35	0.00	92.54	0.00	0.33
RSD	0.07	0.35	0.37	0.35	0.35	0.12	1.11	0.55	1.11

Values represent mean and SD of six determinations.

**(a):** 

Selected parameters and their variations	−1 (lower limit)	+1 (upper limit)
Acetonitrile in mobile phase (%) (*A*)	45	55
Peak modifier (concentration of TEA) (*B*)	200	300
Final pH of the mobile phase (*C*)	2.75	3.25
Flow rate (mL/min) (*D*)	0.9	1.1

**(b):** 

Exp. number	Run order	Factors				Responses		
		*A*	*B*	*C*	*D*	*T*_*r*_	*T*_*f*_	Area
1	13	45	200	2.75	0.9	5.2	1.12	211945
2	14	55	200	2.75	0.9	4.3	1.09	208410
3	8	45	300	2.75	0.9	5.1	1.08	209599
4	10	55	300	2.75	0.9	4.2	1.06	208477
5	3	45	200	3.25	0.9	5.1	1.12	208104
6	2	55	200	3.25	0.9	4.4	1.09	209648
7	1	45	300	3.25	0.9	5.1	1.07	201045
8	7	55	300	3.25	0.9	4.8	1.11	208154
9	12	45	200	2.75	1.1	4.7	1.10	208450
10	11	55	200	2.75	1.1	4.1	1.07	207649
11	5	45	300	2.75	1.1	4.5	1.07	208478
12	4	55	300	2.75	1.1	4.2	1.10	208451
13	9	45	200	3.25	1.1	4.6	1.10	210450
14	16	55	200	3.25	1.1	4.05	1.06	207145
15	15	45	300	3.25	1.1	4.6	1.10	209457
16	6	55	300	3.25	1.1	4.1	1.07	207124

*T*_*r*_ = retention time of drug, *T*_*f*_ = tailing factor for drug, and area count.

**Table 6:** Stability data under different stressed conditions.

Stress conditions	% Trandolapril remained	Relative percentage of degraded products
Oxidative stress (30% H_2_O_2_)	40.9 (peak #2)	13.3 (peak #1), 3.38 (3), 10.05 (4), 26.65 (5), 3.35 (6), and 8.05 (7)

Acidic (0.1 N HCl)	99.6 (peak #2)	0.2 (peak #1) and 0.2 (peak 3)

Alkaline (0.1 N NaOH)	0.0	54.12 (peak #1), 2.22 (2), and 43.66 (3)

Ultraviolet light (2 hours, 80 W)	100.0	0.0

Direct sunlight	100.0	0.0

Aqueous stability (after 21 days)	99.5 ± 0.1	0.0

**Table 7:** Assay of marketed pharmaceutical formulation and API.

Drug/formulation	Present method		BP [18]	
	% Assay	RSD	% Assay	RSD
Capsule	99.2	0.89	98.3	0.95
API	98.9	0.1	98.2	1.2

Student's *t*-test indicates no significant difference (*P* > 0.05).
